# Towards a point-of-care multimodal spectroscopy instrument for the evaluation of human cardiac tissue

**DOI:** 10.1007/s00380-023-02292-3

**Published:** 2023-08-22

**Authors:** Varun J. Sharma, Alexander Green, Aaron McLean, John Adegoke, Claire L. Gordon, Graham Starkey, Rohit D’Costa, Fiona James, Isaac Afara, Sean Lal, Bayden Wood, Jaishankar Raman

**Affiliations:** 1 Department of Surgery, Melbourne Medical School, University of Melbourne, Melbourne, Australia; 2 Brian F. Buxton Department of Cardiac Surgery, Austin Hospital, Melbourne, Australia; 3 Spectromix Laboratory, Melbourne, VIC Australia; 4 Monash Biospectroscopy, Monash University, Melbourne, Australia; 5 Department of Infectious Diseases, Austin Health, Melbourne, VIC Australia; 6 Department of Microbiology and Immunology, The University of Melbourne, The Peter Doherty Institute for Infection and Immunity, Melbourne, VIC Australia; 7 North Eastern Public Health Unit, Austin Health, Melbourne, VIC Australia; 8 Liver Transplant Unit, Austin Hospital, Melbourne, Australia; 9 DonateLife Victoria, Carlton, Melbourne, VIC Australia; 10 Department of Intensive Care Medicine, Melbourne Health, Melbourne, VIC Australia; 11 School of Information Technology and Electrical Engineering, The University of Queensland, Brisbane, Australia; 12 Department of Cardiology, Royal Prince Alfred Hospital, Sydney, Australia; 13 Faculty of Medicine and Health, University of Sydney, Sydney, NSW Australia

**Keywords:** Cardiomyopathy, Vibrational spectroscopy, Point-of-care, Fibrosis

## Abstract

To demonstrate that point-of-care multimodal spectroscopy using Near-Infrared (NIR) and Raman Spectroscopy (RS) can be used to diagnose human heart tissue. We generated 105 spectroscopic scans, which comprised 4 NIR and 3 RS scans per sample to generate a “multimodal spectroscopic scan” (MSS) for each heart, done across 15 patients, 5 each from the dilated cardiomyopathy (DCM), Ischaemic Heart Disease (IHD) and Normal pathologies. Each of the MSS scans was undertaken in 3 s. Data were entered into machine learning (ML) algorithms to assess accuracy of MSS in diagnosing tissue type. The median age was 50 years (IQR 49–52) for IHD, 47 (IQR 45–50) for DCM and 36 (IQR 33–52) for healthy patients (*p* = 0.35), 60% of which were male. MSS identified key differences in IHD, DCM and normal heart samples in regions typically associated with fibrosis and collagen (NIR wavenumbers: 1433, 1509, 1581, 1689 and 1725 nm; RS wavelengths: 1658, 1450 and 1330 cm^−1^). In principal component (PC) analyses, these differences explained 99.2% of the variation in 4 PCs for NIR, 81.6% in 10 PCs for Raman, and 99.0% in 26 PCs for multimodal spectroscopic signatures. Using a stack machine learning algorithm with combined NIR and Raman data, our model had a precision of 96.9%, recall of 96.6%, specificity of 98.2% and Area Under Curve (AUC) of 0.989 (Table 1). NIR and Raman modalities alone had similar levels of precision at 94.4% and 89.8% respectively (Table 1). MSS combined with ML showed accuracy of 90% for detecting dilated cardiomyopathy, 100% for ischaemic heart disease and 100% for diagnosing healthy tissue. Multimodal spectroscopic signatures, based on NIR and Raman spectroscopy, could provide cardiac tissue scans in 3-s to aid accurate diagnoses of fibrosis in IHD, DCM and normal hearts.

**Table 1:** Machine learning performance metrics for validation data sets of (a) Near-Infrared (NIR), (b) Raman and (c and d) multimodal data using logistic regression (LR), stochastic gradient descent (SGD) and support vector machines (SVM), with combined “stack” (LR + SGD + SVM)

	AUC	Precision	Recall	Specificity
(a) NIR model				
Logistic regression	0.980	0.944	0.933	0.967
SGD	0.550	0.281	0.400	0.700
SVM	0.840	0.806	0.800	0.900
Stack	**0.933**	**0.794**	**0.800**	**0.900**
(b) Raman model				
Logistic regression	0.985	0.940	0.929	0.960
SGD	0.892	0.869	0.857	0.932
SVM	0.992	0.940	0.929	0.960
Stack	**0.954**	**0.869**	**0.857**	**0.932**
(c) MSS: multimodal (NIR + Raman) to detect DCM vs. IHD vs. normal patients				
Logistic regression	0.975	0.841	0.828	0.917
SGD	0.847	0.803	0.793	0.899
SVM	0.971	0.853	0.828	0.917
Stack	**0.961**	**0.853**	**0.828**	**0.917**
(d) MSS: multimodal (NIR + Raman) to detect pathological vs. normal patients				
Logistic regression	0.961	0.969	0.966	0.984
SGD	0.944	0.967	0.966	0.923
SVM	1.000	1.000	1.000	1.000
Stack	**1.000**	**0.944**	**0.931**	**0.969**

Bold values indicate values obtained from the stack algorithm and used for analyses

Machine learning performance metrics for validation data sets of (a) Near-Infrared (NIR), (b) Raman and (c and d) multimodal data using logistic regression (LR), stochastic gradient descent (SGD) and support vector machines (SVM), with combined “stack” (LR + SGD + SVM)

Bold values indicate values obtained from the stack algorithm and used for analyses

## Background

Heart disease is the leading cause of hospitalisations, morbidity and mortality globally, but there are no point-of-care instruments that can provide metabolic or morphological diagnoses in real time. Current diagnostic modalities, such as angiograms, echocardiograms, magnetic resonance imaging or nuclear medicine scans, are either invasive or provide only anatomical assessments. They are poorly sensitive to molecular and metabolic (cellular activity) changes. These assessments can only be achieved using endo-myocardial biopsies (EMB), which are fraught with life-threatening operative risks, are personnel and resource intensive and are therefore rarely used clinically [1]. This represents a significant unmet need, as the lack of a point-of-care instrument to detect morphology and metabolic activity has significant diagnostic and prognostic implications on patient treatment. In Ischaemic heart disease, the leading cause of mortality globally, identifying “viability” or reversible ischaemia morphology could identify patients that would benefit from surgery to re-vascularize this tissue [2, 3]. In heart failure, the leading cause of hospitalisations globally, morphological and metabolic assessment could crucially identify the underlying cause heart failure, which is especially important when the aetiology is unknown or treatable with medical therapy (e.g. dilated cardiomyopathy, restrictive cardiomyopathy, such as amyloidosis, sarcoidosis, hypersensitivity myocarditis, anthracycline cardiomyopathy, tumours and arrhythmogenic right ventricular cardiomyopathy). In heart transplantation, there is potential to increase the number of hearts available for transplantation, as a rapid assessment of donor heart morphology can identify potentially usable hearts that would otherwise be rejected. Furthermore, for patients who are peri- or post-transplant, it could provide a non-invasive method of monitoring the progression of disease of heart tissue. Currently, evaluation of rejection of transplanted hearts relies on routine EMB. The absence of a point-of-care instrument is therefore an unmet need that significantly amplifies the burden of cardiovascular disease globally. 

We believe this unmet need can be addressed by combining advancements in vibrational spectroscopy (Raman Spectroscopy, RS; Near Infra-Red Spectroscopy, NIRS; and mid infrared spectroscopy, MIRS) with computational methods (machine learning). These technologies have potential to provide non-perturbative, rapid and label-free tissue assessment of morphology and metabolism [4–7] at the molecular level [8, 9]. In the cardiovascular space, studies from animal or preserved tissue [4, 7, 10–16] demonstrate that these techniques can quantify myocardial fibrosis by exploring collagen subtypes [17–20], cross linking [21, 22] and distribution [20, 23]. The techniques are complementary, with RS techniques being more sensitive to metabolic data at a molecular level [24], and infrared-based techniques (such as MIRS, NIRS) better equipped to assess morphological changes [24]. Advances in machine learning have significantly reduced the processing time for analysing these data, and bear potential for real-time diagnoses. Clinical translation is hindered by current studies being restricted to one modality of spectroscopy (RS, Mid IR or NIRS) and have not yet used point-of-care instruments on human tissue.

In this report, we combine RS and NIRS scans of cardiac tissue to obtain non-invasive multimodal spectroscopic signatures (MSS) and use machine learning (ML) to compare its accuracy to conventional histopathology. These scans, which can be performed in 3 s, are iterative steps to generating a point-of-care and non-invasive morphological and metabolic diagnoses in heart disease.

## Methods

### Ethics statement

This study was approved by the Human Resources and Ethics committee (HREC) at Austin Hospital, Heidelberg, Melbourne, Victoria (HREC/73660/Austin-2021). Approval of the acquisition of human tissue from organ donors was as part of the Australian Donation and Transplantation Biobank (HREC/4814/Austin-2019) and Donate Life Victoria (DLV) through the Australian Red Cross Lifeblood Health Human Research and Ethics Committee (Ethics 2019#08). Human tissue from explanted hearts was with USYD HREC 2021/122. Individual consent was obtained from either the patient or Senior Next Available next of Kin (SANOK) prior to accessing samples. Ethics approvals are in keeping with the Declaration of Helsinki.

### Sample retrieval

Pathological human heart samples were obtained from the Sydney Heart Bank at the University of Sydney, New South Wales, Australia [25] from patient samples of dilated cardiomyopathy and ischaemic heart disease pathologies. Physiological control patient samples were prospectively collected from consecutive donors providing organs for transplantation in Victoria, Australia from the Australian Donation and Transplantation Biobank (ADTB) [26]. These were taken from hearts not utilised in transplantation, with the protocol having been described previously [26, 27]. For analysis, all fresh samples were thawed under standard laboratory conditions. All samples were de-identified and anonymously catalogued at the time of excision and source blinded for all subsequent analysis.

### Multimodal spectroscopic signatures

**Fig. 1:**
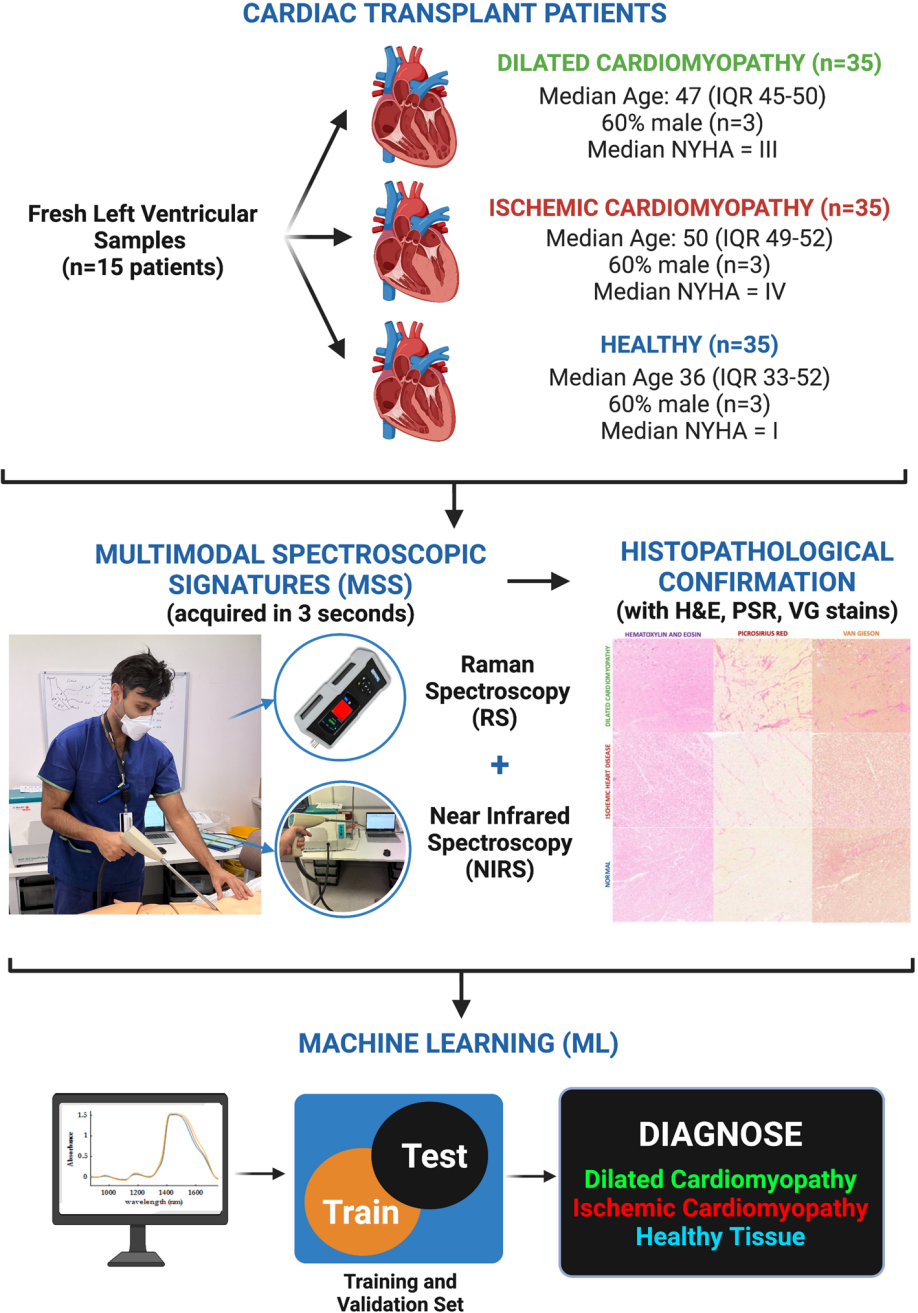
Algorithm for Multimodal Spectroscopic Signature acquisition from cardiac patient samples. Fresh tissue was acquired from 15 patients at time of cardiac transplantation, either from explanted dilated and ischaemic cardiomyopathy patients or healthy hearts at time of organ retrieval. Samples were scanned using separate Raman (3 scans per sample) and NIRS (4 scans per sample) instruments to acquire an MSS (combines the 7 scans per sample, 35 in total for each pathology), and then entered into machine learning (ML) algorithms to assess their diagnostic accuracy. *H&E* haematoxylin and eosin, *PSR* picrosirius red, *VG* Van Gieson. Created with BioRender.com

We acquired multimodal spectroscopic signatures from all fresh patient samples using near-infrared and Raman spectroscopy (Fig. 1). All scans were undertaken using commercially available handheld point-of-care instruments, with a combined scanning time of 3 s. NIRS spectral of fresh patient samples were acquired with Metrohm NIRS S XDS Interactance OptiProbe Analyzer (Metrohm, Herisau Switzerland; wavelength range 400–2500 nm). Raman measurements were taken using TacticID-1064 ST (Metrohm, Herisau Switzerland; wavenumber range 2500–176 cm^−1^). Patient samples were thawed with overnight refrigeration at − 4 °C, and then thawed at room temperature 4 h prior to analysis. Spectral acquisition was performed using the inbuilt Metrohm Software. The duration of each of the MSS scans was 3 s and comprised 4 NIRS and 3 RS scans per sample. To ensure each MSS scan was a holistic spectral representation of the underlying tissue, the positions for each of the 7 scans per sample were selected at random. Each of the 15 samples was analysed separately and labelled to ensure accurate histological correlations. Histological evaluations were performed of the tissues at the points of where the spectroscopic scans were performed. Instruments were positioned perpendicular to the tissue (Fig. 1) by hand, with complete apposition of the tissue with the probe surface to prevent infiltration of surrounding light.

### Histopathological confirmation

Results from MSS scans were compared to the gold standard of histopathology. After analysis of fresh samples by MSS, each of the 15 samples were separately embedded in paraffin, sectioned and stained with Haematoxylin and Eosin, Picrosirius Red (PSR) and Van Geison’s (VG) stains for assessment of fibrosis. Samples were diagnosed by a qualified Anatomical Pathologist to confirm the explant diagnoses, classified as either (a) Dilated Cardiomyopathy, (b) Ischaemic Cardiomyopathy or (c) Healthy tissue.

### Data analysis

Study data were collected and managed using Research Electronic Data Capture (REDCap) electronic data capture tools hosted at ADTB. Analysis of clinical data was done using Stata v15.0 (StataCorp. 2017. Stata Statistical Software: Release 15. College Station, TX: StataCorp LLC), with clinical variables reported as either counts with corresponding percentages, or median averages with interquartile range (IQR).

### Machine learning

**Fig. 2:**
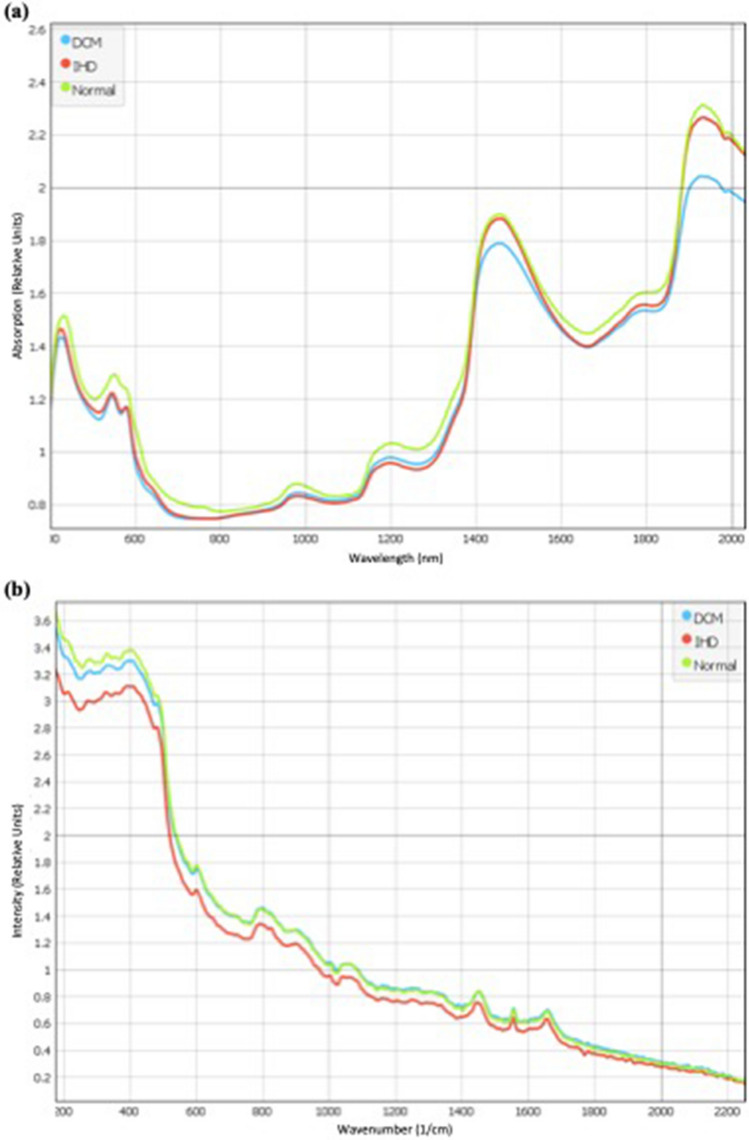
Average spectroscopic signatures (i.e. not pre-processed) for dilated cardiomyopathy (blue), ischaemic heart disease (red), and healthy tissue (green) using **a** near-infrared spectroscopy (NIRS) and **b** Raman spectroscopy (RS)

NIRS and RS data (Fig. 2) were entered into a pre-processing algorithm using Quasar [28], which included keeping a region of spectra, and a Savitzky–Golay filter to ensure normalised and baseline corrected data. The pre-processing for NIRS was undertaken using previously established protocols [28], by keeping 600–1750 nm, Savitzky–Golay filter (window = 15, polynomial order = 2, derivative order = 2) and area normalisation peak from 0. The pre-processing for RS was done by keeping 600–1750 nm, Savitzky–Golay filter (window = 19, polynomial order = 2, derivative order = 2), SNV normalisation and baseline correction. The data from each of the 4 NIRS and 3 RS scans were combined for each sample, to generate one MSS per patient (i.e. 15 MSS in total). Principal Component (PC) analysis was used to identify potential model outliers. Pre-processed data were then entered into Logistic Regression (LR), Support Vector Machine (SVM) and Stochastic Gradient Descent (SGD) machine learning (ML) algorithms [29, 30]. LR had regularisation type Lasso (L1) and strength C = 3. SVM used v-SVM with regression cost = 1.0, complexity bound = 0.5, RBF Kernel with g = auto, numerical tolerance of 0.01 and iteration limit of 100. SGD used an optimal learning rate with 1000 iterations and a tolerance of 0.001, Lasso (L1) regularisation with strength 0.01, and the Loss function and was classified by squared loss with Huber regression at 0.1. The parameters for these models are continuously used across NIRS and RS data. The “stack” ML algorithm uses a boosting technique to incrementally combine all individual methods (LR, SGD, SVM), and data for NIRS, RS and MSS are reported separately. To avoid overfitting, the validation dataset was obtained by compiling averages of the technical replicates acquired from each sample. This was set to avoid overfitting of models thereby avoiding the “technical replicate trap” [31]. Four metrics on the validation dataset are presented, with area under the receiver-operator curve (AUC) providing an aggregate measure of performance across all possible classification thresholds, precision measuring rate of detecting positives across all true positives, recall measuring true positives amongst those who test positive, and sensitivity measuring true negative amongst those true negatives. The diagnostic capability of the model was assessed using confusion matrices with classification rates.

## Results

### Demographics

We generated 105 spectroscopic scans, which comprised 4 NIR and 3 RS scans per sample to generate a “multimodal spectroscopic signature” for each heart, done across 15 patients, 5 each from the dilated cardiomyopathy (DCM), Ischaemic Heart Disease (IHD) and Non-Diseased pathologies. These were compared to the gold standard of histopathology in final analysis. The median age of patients was 49 years (IQR 43–52), of which 60% (*n* = 9/15) were male, with stratification based on disease shown in Fig. 1. The averaged NIRS (Fig. 2a) and RS (Fig. 2b) spectra for each disease subtype are shown in Fig. 2. On visual assessment, MSS showed variation in the wavenumber values typically associated with cardiac fibrosis [32] and collagen [33, 34]. In the NIRS (Fig. 2a), these were bands at 1433, 1509, 1581, 1689, and 1725 nm, and in Raman (Fig. 2b), these were at 1658, 1450 and 1330 cm^−1^. Band assignments are listed in the supporting information.

### Principal component analysis

**Fig. 3:**
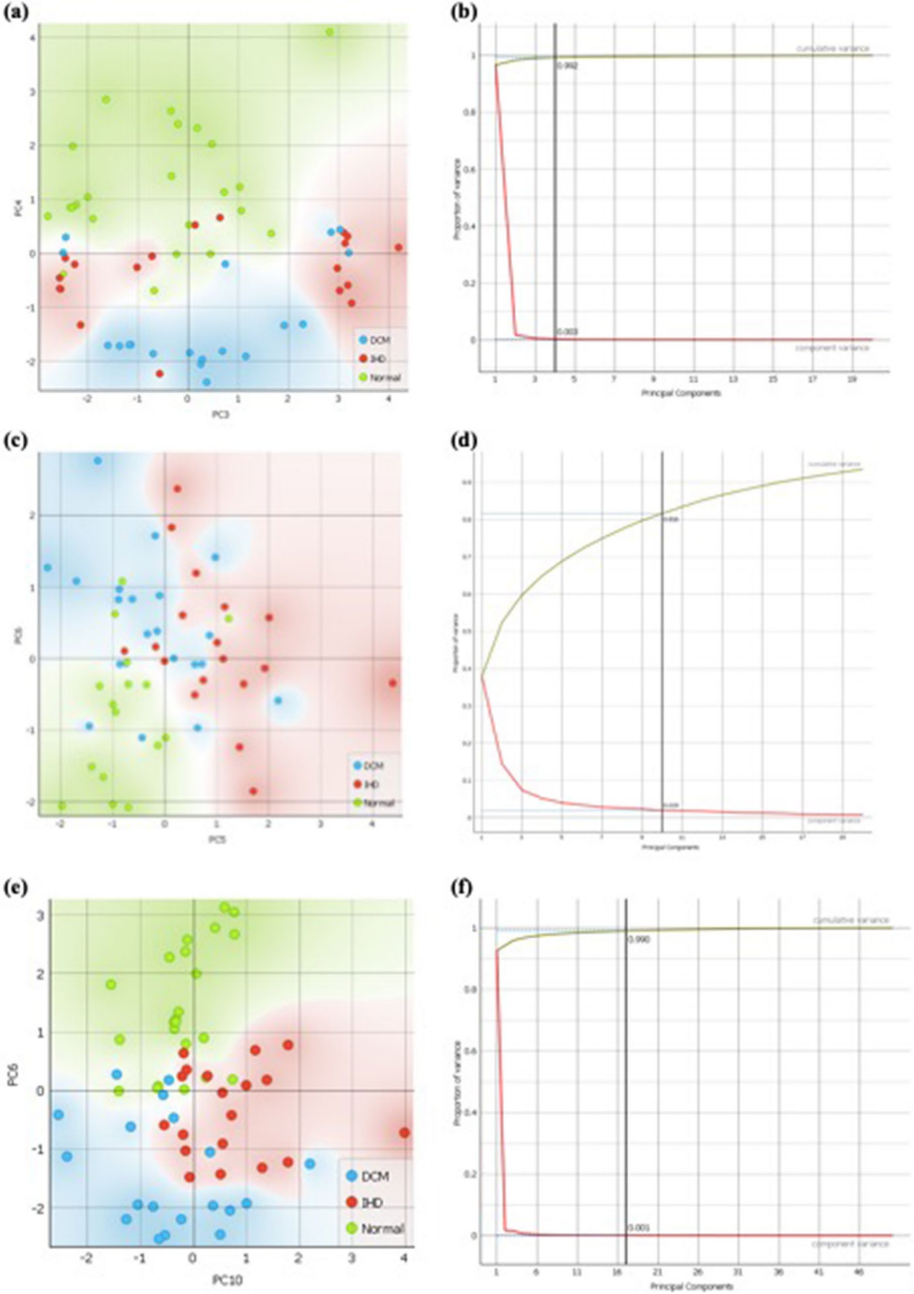
Principal component analysis showing **a** NIR scatter plot with **b** PCA components plot; **c** Raman scatter plot with **d** ROC curve; **e** multimodal scatter plot combined with **f** ROC curve

We aimed to demonstrate that variation in chemical signatures between DCM, IHD and normal patients could be attributed to the differences visualised in absorption and scattering at key regions (or Principal Components, PC) as identified above (Fig. 2). PC analysis is demonstrated in Fig. 3, where we differentiate the three disease classes (IHD, DCM and Normal) using near-infrared (NIRS; Fig. 3a and b), Raman (RS; Fig. 3c and d) and multimodal (MSS; Fig. 3e and f) spectral profiles. The corresponding loadings plots are shown in the appendix. The NIRS data demonstrated best discrimination, with 99.2% explained variance (Fig. 3b) using 4 principal components (PC). The visual scores plot (Fig. 3a) demonstrates that healthy (Normal) patients are discriminated from diseased (IHD and DCM) patients along PC 4 (y axis), where the PC 3 (x axis) then further discriminates IHD from DCM. Raman data demonstrated 81.6% of explained variance with 10 components and complements NIR data. Healthy patients are well discriminated in both PC 5 and 6, where PC 5 discriminates the disease subtypes (IHD vs. DCM) best. In MSS analysis (NIR and Raman), we demonstrate 99.0% of explained variance in 26 components, where a plot between PC6 and PC10 stratifies the three disease classes (IHD vs. DCM vs. Normal) best. This demonstrates that most of the variation (81.6–99.2%) between disease types can be attributed to a series of key components (PCs) in our chemical signatures.

After identifying outliers and testing the robustness of our data through Principal component analysis, we entered NIR, Raman and Multimodal data into Logistic Regression, Stochastic Gradient Descent (SGD) and Support Vector Machine (SVM) Machine Learning algorithms and then a combined (stack) algorithm. Using a multimodal technique (NIR + Raman) and combining ML techniques, our model had a precision of 85.3%, recall of 82.8%, specificity of 91.7% and AUC of 0.961 in distinguishing between IHD, DCM and Normal disease types. When simplified into determining Pathological (DCM and IHD) from Normal patient samples, our model had a 94.4%, recall of 93.1%, specificity of 96.9% and AUC of 1.00.

### Confusion matrices

**Fig. 4:**
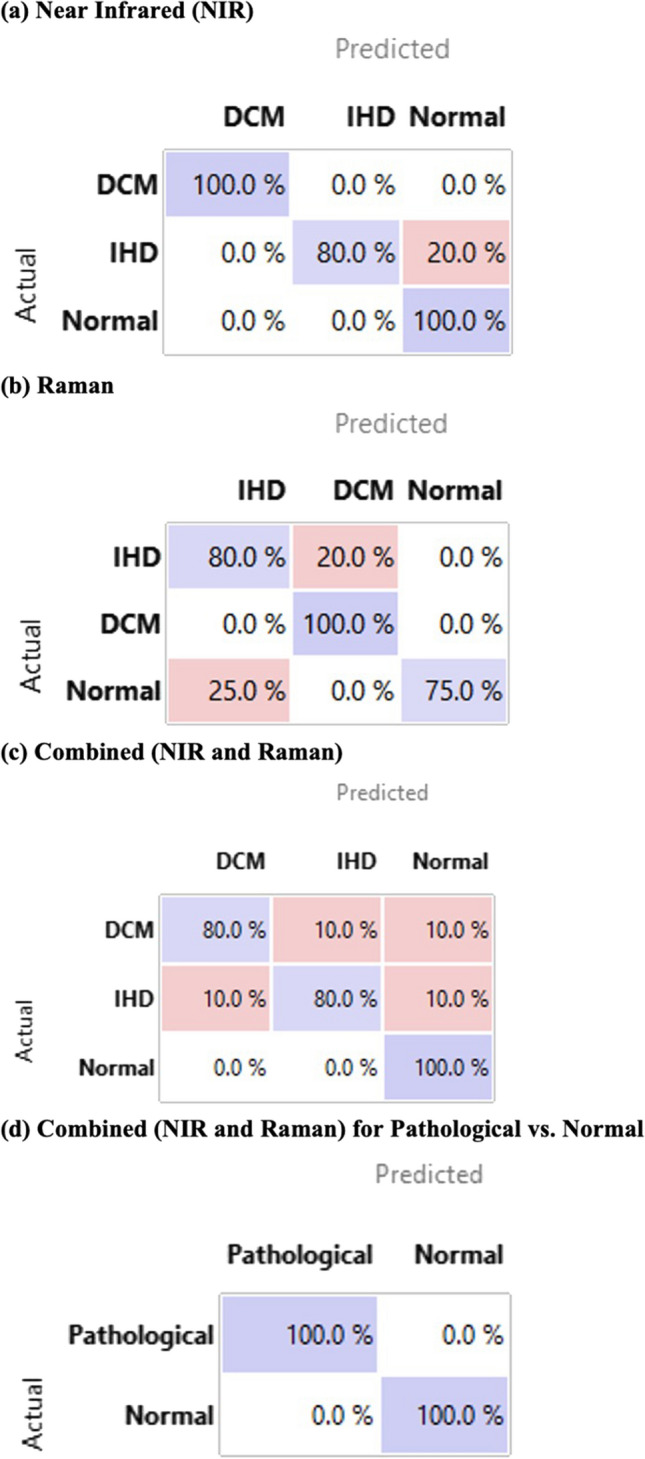
Confusion Matrices for Stack machine algorithm, where the predicted (x axis) are plotted against actual (y axis) based on **a** near-infrared spectra, **b** Raman spectra and **c** combined NIR and Raman spectra

Using the stack machine learning model, we plotted confusion matrices (Fig. 4) where MSS’ predictions (Predicted) are plotted against the true (Actual) classifications. The multimodal approach demonstrated a classification accuracy of 80% for detecting dilated cardiomyopathy, 80% for ischaemic heart disease and 100% for diagnosing healthy patient samples. When simplified into an instrument to detect Normal vs. Pathological (IHD and DCM), we observe 100% classification accuracy. Sub-stratifications based on modality (NIR and RS) are shown in Fig. 4.

## Discussion

To our knowledge, this is the first multi-modal spectroscopic analysis of human cardiac tissue. We have used a set of hand-held instruments with point-of-care capacity to generate multimodal spectroscopic signatures (MSS), which are done in 3-s, and used it to diagnose cardiac disease. We derive the following key findings. First the key differences in MSS of IHD, DCM and healthy patients are in regions associated with myocardial fibrosis and collagen deposition. This is consistent with our observations in principal component analysis, where a handful of key differences (principal components, PC) in the MSS explains the majority of variance. In NIRS, we can explain 99.2% of the variance in 4 PCs; in RS 81.6% with 10 PCs; and 99.0% with 26 PCs in the final combined MSS scans (NIR + Raman). When deployed as a machine learning (ML) model, MSS had excellent accuracy, with a precision of 94.4%, recall of 93.1%, and specificity of 96.9% and AUC of 1.00. When implemented as a diagnostic instrument, MSS demonstrated a classification accuracy of 100% in identifying abnormal patients. 

This adds to the literature in providing the next iterative step in providing a point-of-care diagnostic tool for cardiac tissue in a non-invasive manner. So far, the closest rendition has been the assessment by Adegoke et al. [35] who demonstrated good discrimination of NIR in identifying murine renal fibrosis. In the present study, fresh frozen tissue from human patients was used, where there were definitive histopathological diagnoses: samples were either end-stage heart disease patients who had hearts explanted (IHD or DCM), or healthy tissue from organ donors. We provide a large number of spectra (*n* = 105) of fresh human tissue. Human tissue that is unprocessed, has been historically challenging to access. As a consequence, studies to date have been on animal or preserved tissue. Lastly, fresh tissue that has not been subject to conventional processing for histopathology was scanned and analysed, thereby emulating in vivo use. These findings are also consistent with emerging data that have shown spectroscopy in assessing cardiac tissue, albeit with bulky laboratory instruments [13, 33, 34]. We validate these findings using a range of ML techniques (Logistic Regression, SGD, and SVM) and find that the “stack” algorithm in the multimodal models does not vary drastically from the results of the individual ML models (Table 1). Although analyses in this study were done post hoc, we believe that advancements in ML will enable to be this done in an instantaneous manner in the future iterations. 

A point-of-care instrument that is able to diagnose underlying morphology has significant diagnostic and prognostic implications on patient treatment. In ischaemic heart disease, the leading cause of mortality globally, identifying “viability” or reversible ischaemia can identify patients that would benefit from surgery to re-vascularize this tissue [2, 3]. In heart failure, the leading cause of hospitalisations globally, morphological and metabolic assessment can crucially identify the underlying cause heart failure, which is especially important when the aetiology is unknown or treatable with medical therapy (e.g. dilated cardiomyopathy, restrictive cardiomyopathy, such as amyloidosis, sarcoidosis, hypersensitivity myocarditis, anthracycline cardiomyopathy, tumours and arrhythmogenic right ventricular cardiomyopathy). In heart transplantation, one could potentially increase the number of hearts available for transplantation, as a rapid assessment of donor heart molecular or metabolic changes can identify potentially usable hearts that would otherwise be rejected. This may have implications in xenotransplantation as well [36]. Furthermore, for patients who are peri- or post-transplant, it can provide a non-invasive method of monitoring the progression of disease or heart tissue; a method that currently relies on routine EMB. These would need catheter-based adaptation of the technology [37]. A point-of-care instrument can therefore significantly reduce the economic, quality-adjusted life years (QALY) and mortality of heart disease globally.

The weaknesses of this study are as follows: Due to the scarcity and challenging nature of obtaining these tissues, we have only been able to obtain multiple samples from 15 patients which allows us to present preliminary data, and had to use a validation dataset that represents an average of technical replicates. This may lend itself to over-optimistic results, but we are undertaking larger studies in order to improve its robust diagnostic capability. To negate this, we undertook several ML techniques (LR, SGD, SVM), and even though results for SVM were superior to other techniques, present results from the “stack” algorithm as a method of increasing the number of methods by which our dataset has been validated. This study was not powered enough for higher level functions, such as quantifying percentage of fibrosis, or diagnosing subtypes of disease patterns, aetiology of dilated cardiomyopathy or pattern of hypertrophy for ischaemic heart disease. Larger scale studies will have the capacity to do this. Furthermore, Raman data only makes marginal improvements to the performance of our model, and the 26 PCs of RS are less reliable than the 4 PCS of NIRS. However, adding Raman data is an important iterative step in demonstrating its capacity for assessing human cardiac tissue which has so far not been done in any study to our knowledge. In an era where ML techniques can make instantaneous predictions using advanced computing, this hurdle be easily overcome once the robustness of MSS is established, which we hope these findings help establish. 

## Data Availability

The datasets used and analyzed during the current study are available from the corresponding author on reasonable request.
